# Hematologic and metabolic indices for predicting 28-day mortality in sepsis patients: a retrospective intensive care cohort study

**DOI:** 10.3389/fmed.2026.1833902

**Published:** 2026-05-11

**Authors:** Sami Uyar, Hatice Eyiol, Ahmet Yılmaz, Azmi Eyiol, Yakup Alsancak

**Affiliations:** 1 Department of Anesthesiology and Reanimation, Beyhekim Training and Research Hospital, Konya, Türkiye; 2 Department of Cardiology, Faculty of Medicine, Karamanoğlu Mehmetbey University, Karaman, Türkiye; 3 Department of Cardiology, Beyhekim Training and Research Hospital, Konya, Türkiye; 4 Department of Cardiology, Necmettin Erbakan University, Konya, Türkiye

**Keywords:** biomarkers, HRR, intensive care unit, mortality, RAR, sepsis, TyG index, uric acid-to-albumin ratio

## Abstract

**Background:**

Sepsis remains a leading cause of mortality in critically ill patients, and early risk stratification is essential for improving clinical outcomes. Recently, hematological and metabolic indices derived from routine laboratory parameters have emerged as potential prognostic biomarkers. This study aimed to evaluate the predictive value of the hemoglobin-to-red cell distribution width ratio (HRR), red cell distribution width-to-albumin ratio (RAR), triglyceride–glucose (TyG) index, and uric acid-to-albumin ratio (UA/Alb) for 28-day mortality in patients with sepsis.

**Methods:**

This retrospective cohort study included 805 adult patients with sepsis admitted to the intensive care units of two tertiary centers between January 2020 and September 2025. Demographic data, clinical severity scores (qSOFA, SOFA, APACHE II, SAPS II), and laboratory parameters obtained within the first 24 h of ICU admission were analyzed. The primary outcome was 28-day all-cause mortality. Receiver operating characteristic (ROC) curve analysis was performed to assess discriminative performance, and logistic regression analyses were conducted to identify independent predictors of mortality.

**Results:**

Non-survivors had significantly higher disease severity scores and exhibited a distinct biochemical profile characterized by lower hemoglobin and albumin levels and higher RDW, uric acid, triglyceride, and glucose levels (all *p* < 0.001). All evaluated indices were significantly associated with mortality. The UA/Alb ratio showed the highest predictive performance (AUC: 0.968), followed by the TyG index (AUC: 0.916) and RAR (AUC: 0.900), while HRR demonstrated moderate discrimination (AUC: 0.771). In multivariable analysis, the UA/Alb ratio (OR: 28.47, 95% CI: 12.96–62.51; *p* < 0.001) and SAPS II score remained independent predictors of 28-day mortality.

**Conclusion:**

Hematological and metabolic indices are strongly associated with and may improve early prediction of mortality in septic patients. Among these, the UA/Alb demonstrated the highest discriminative performance in this cohort and remained associated with mortality after adjustment; however, its independence from established severity scores should be interpreted with caution.

## 1 Introduction

Sepsis is a life-threatening condition characterized by a dysregulated host response to infection leading to organ dysfunction and high mortality, particularly among critically ill patients admitted to intensive care units (ICUs). Despite advances in supportive care and antimicrobial therapy, sepsis remains a major global health problem with significant morbidity and mortality. Early identification of high-risk patients and timely risk stratification are essential for improving clinical outcomes and guiding therapeutic strategies. Current international guidelines emphasize the importance of early recognition, hemodynamic stabilization, and continuous monitoring in the management of sepsis and septic shock. However, identifying reliable and easily accessible biomarkers for predicting prognosis in septic patients remains a significant clinical challenge. Therefore, increasing attention has been directed toward simple laboratory-derived indices that may assist in early risk assessment and mortality prediction in critically ill patients with sepsis (1–5).

The hemoglobin-to-red cell distribution width ratio (HRR) has recently emerged as a novel hematological index reflecting both oxygen-carrying capacity and underlying inflammatory or nutritional status. Hemoglobin levels may decrease due to inflammation-related anemia, while red cell distribution width (RDW) increases in the presence of systemic inflammation, oxidative stress, and impaired erythropoiesis. The combination of these two parameters in the HRR provides a composite marker that may better reflect disease severity compared to individual parameters alone. Recent studies using large critical care databases have demonstrated that a lower HRR is associated with increased mortality in patients with sepsis and other critical illnesses. These findings suggest that HRR may serve as a practical and readily available prognostic biomarker in septic patients admitted to the ICU (6–8).

Another promising marker is the red cell distribution width-to-albumin ratio (RAR), which integrates RDW, an indicator of systemic inflammation and erythrocyte dysfunction, with serum albumin, a marker reflecting nutritional status, inflammatory burden, and disease severity. Hypoalbuminemia is frequently observed in critically ill patients due to increased vascular permeability, decreased hepatic synthesis, and systemic inflammatory responses. The RAR has therefore been proposed as a composite index representing both inflammatory and nutritional aspects of critical illness. Recent retrospective cohort studies have reported significant associations between elevated RAR levels and increased mortality among septic patients in intensive care settings, highlighting its potential value as a prognostic biomarker (9–12).

Metabolic dysregulation also plays an important role in the pathophysiology of sepsis. The triglyceride-glucose (TyG) index, which is derived from triglyceride and glucose levels, has been widely recognized as a surrogate marker of insulin resistance and metabolic stress. In critically ill patients, metabolic alterations including hyperglycemia and lipid metabolism disturbances frequently occur due to inflammatory and stress responses. Several recent studies have demonstrated that an elevated TyG index is associated with increased mortality, organ dysfunction, and adverse outcomes in septic patients. These findings suggest that the TyG index may serve as an accessible metabolic indicator for identifying high-risk septic patients in the ICU (13–18).

More recently, the uric acid-to-albumin ratio (UAR) has been investigated as a novel biomarker reflecting oxidative stress, systemic inflammation, and nutritional status. Uric acid levels increase in response to tissue hypoxia and oxidative stress, whereas albumin possesses antioxidant and anti-inflammatory properties. The combined evaluation of these two parameters may therefore provide additional prognostic information in critically ill patients. Emerging evidence suggests that elevated UAR levels are associated with worse outcomes and increased mortality in patients with sepsis and other inflammatory conditions. Consequently, the UAR has gained attention as a potential biomarker for risk stratification in critically ill septic patients (19–21).

Therefore, the present study aimed to investigate the prognostic value of several hematological and metabolic ratios, including the hemoglobin-to-red cell distribution width ratio (HRR), red cell distribution width-to-albumin ratio (RAR), triglyceride–glucose (TyG) index, and uric acid-to-albumin ratio (UAR), for predicting 28-day mortality in patients with sepsis admitted to the intensive care unit. Using a retrospective cohort design, we sought to evaluate the association between these easily obtainable laboratory-derived indices and short-term mortality outcomes. Furthermore, we aimed to determine whether these markers could serve as practical and accessible tools for early risk stratification in critically ill patients with sepsis.

## 2 Materials and methods

### 2.1 Study design and population

This study was designed as a retrospective observational cohort study. The study population consisted of adult patients diagnosed with sepsis who were admitted to the intensive care units (ICUs) of Necmettin Erbakan University Meram Faculty of Medicine Hospital and Konya Beyhekim Training and Research Hospital between January 1, 2020 and September 30, 2025.

A total of 805 patients meeting the study criteria were included in the analysis. Demographic characteristics, clinical severity scores at ICU admission, laboratory findings, and clinical outcomes were obtained from electronic medical records and hospital information systems.

The study protocol was approved by the Necmettin Erbakan University Non-Drug and Non-Medical Device Research Ethics Committee (Approval No: 2025/6113, Meeting No: 229, Date: 14 November 2025), and the study was conducted in accordance with the principles of the Declaration of Helsinki. Due to the retrospective design of the study, the requirement for informed consent was waived.

### 2.2 Data collection

For each patient, demographic data including age and sex were recorded. In addition, clinical severity scores assessed at ICU admission were collected, including the quick Sequential Organ Failure Assessment (qSOFA) score, Sequential Organ Failure Assessment (SOFA) score, Acute Physiology and Chronic Health Evaluation II (APACHE II) score, and Simplified Acute Physiology Score II (SAPS II).

Laboratory parameters obtained within the first 24 h after ICU admission were analyzed. These included complete blood count parameters and biochemical measurements such as hemoglobin, red cell distribution width (RDW), triglyceride, glucose, uric acid, and albumin levels.

Based on these laboratory data, several hematological and metabolic indices were calculated:

- Hemoglobin-to-red cell distribution width ratio (HRR) = Hemoglobin/RDW.
- Red cell distribution width-to-albumin ratio (RAR) = RDW/Albumin.
- Triglyceride-glucose (TyG) index = ln [Triglyceride (mg/dL) × Glucose (mg/dL)/2].
- Uric acid-to-albumin ratio (UA/Alb) = Uric acid/Albumin.

These indices were evaluated for their association with 28-day mortality in septic patients admitted to the ICU.

### 2.3 Inclusion criteria

Patients were included in the study if they met the following criteria:

- Age ≥18 years.
- Diagnosis of sepsis according to the Sepsis-3 criteria.
- Availability of baseline laboratory parameters (complete blood count and biochemical tests) obtained within the first 24 h after ICU admission.

### 2.4 Exclusion criteria

Patients were excluded if they met any of the following conditions:

- Pregnancy.
- Presence of active hematological malignancy.
- Patients receiving chronic hemodialysis.
- Presence of a do-not-resuscitate (DNR) order at ICU admission.
- Patients with incomplete laboratory parameters required for index calculation were excluded during the initial data preparation phase.
- Outcome Measure.

After applying the exclusion criteria and data cleaning procedures, 805 patients with complete variables required for the final analyses were included.

The primary outcome of the study was 28-day all-cause mortality. Patients were classified into survivor and non-survivor groups based on their survival status within 28 days following ICU admission.

### 2.5 Statistical analysis

All statistical analyses were performed using IBM SPSS Statistics v.26.0 (IBM Corp., United States) and R software (v.4.3.1; R Foundation for Statistical Computing, Vienna, Austria). Continuous variables were assessed for normality using the Kolmogorov–Smirnov test and visual inspection of histograms.

Continuous variables were presented as mean ± standard deviation (SD) for normally distributed data and as median with interquartile range (IQR) for non-normally distributed variables. Categorical variables were expressed as numbers and percentages. Comparisons between survivor and non-survivor groups were performed using the Student’s t-test or Mann–Whitney U test for continuous variables, depending on data distribution, and the chi-square test or Fisher’s exact test for categorical variables.

To evaluate the prognostic performance of the hematological and metabolic indices, receiver operating characteristic (ROC) curve analysis was performed and the area under the curve (AUC) was calculated with 95% confidence intervals (CI). The optimal cut-off values were determined using the Youden index.

Univariate logistic regression analysis was initially performed to identify variables associated with 28-day mortality. Variables showing statistical significance in univariate analysis were subsequently included in a multivariate logistic regression model to determine independent predictors of 28-day mortality. Results were presented as odds ratios (ORs) with 95% confidence intervals (CIs).

In addition, Spearman or Pearson correlation analysis was performed to evaluate the relationships between the studied indices (HRR, RAR, TyG, and UA/Alb) and clinical severity scores (SOFA, APACHE II, and SAPS II).

Internal validation was performed using bootstrap resampling (1,000 iterations) and 10-fold cross-validation.

A two-tailed *p*-value < 0.05 was considered statistically significant.

## 3 Results

A total of 805 patients with sepsis admitted to the intensive care unit were included in the analysis. Patients were stratified according to 28-day mortality status, of whom 228 (28.3%) were survivors and 577 (71.7%) were non-survivors. Non-survivors were significantly older than survivors, with a median age of 79.0 (72.0–85.0) years compared to 73.5 (64.0–80.0) years in survivors (*p* < 0.001). There was no statistically significant difference between groups in terms of sex distribution (*p* = 0.726; Table 1).

**Table 1:** Baseline characteristics of the study population according to 28-day mortality.

Variable	Survivors (*n* = 228)	Non-survivors (*n* = 577)	*p*-value
Age, years	73.5 (64.0–80.0)	79.0 (72.0–85.0)	<0.001
Sex, male, *n* (%)	134 (58.8%)	330 (57.2%)	0.726
qSOFA score	1.0 (0.0–2.0)	3.0 (3.0–3.0)	<0.001
SOFA score	6.0 (4.0–8.0)	12.0 (11.0–13.0)	<0.001
APACHE II score	18.0 (14.0–22.0)	57.0 (50.0–70.0)	<0.001
SAPS II score	35.0 (29.0–41.0)	65.0 (52.0–78.0)	<0.001
Hemoglobin, g/dL	12.2 (10.8–13.7)	9.9 (8.9–11.9)	<0.001
RDW, %	14.5 (13.5–15.6)	17.0 (15.3–19.0)	<0.001
Albumin, g/L	36.0 (32.0–40.0)	25.5 (23.0–28.7)	<0.001
Uric acid, mg/dL	6.6 (6.0–7.1)	7.8 (7.3–8.5)	<0.001
Triglyceride, mg/dL	111.0 (95.0–128.0)	155.0 (137.0–188.0)	<0.001
Glucose, mg/dL	99.0 (89.0–113.0)	137.0 (117.5–193.5)	<0.001
HRR (hemoglobin/RDW)	0.83 (0.72–0.97)	0.58 (0.48–0.75)	<0.001
RAR (RDW/albumin)	0.40 (0.35–0.48)	0.66 (0.56–0.78)	<0.001
TyG index	8.48 (8.23–8.73)	9.32 (9.03–9.74)	<0.001
UA/Alb ratio	0.18 (0.15–0.21)	0.29 (0.26–0.35)	<0.001

Data are presented as median (interquartile range, IQR) for continuous variables and number (percentage) for categorical variables. Comparisons between 28-day survivors and non-survivors were performed using the Mann–Whitney U test for continuous variables and the *χ*^2^ (chi-square) test for categorical variables. All p values are two-sided, and *p* < 0.05 was considered statistically significant. Abbreviations: qSOFA, quick Sequential Organ Failure Assessment; SOFA, Sequential Organ Failure Assessment; APACHE II, Acute Physiology and Chronic Health Evaluation II; SAPS II, Simplified Acute Physiology Score II; RDW, red cell distribution width; HRR, hemoglobin-to-red cell distribution width ratio; RAR, red cell distribution width-to-albumin ratio; TyG, triglyceride-glucose index; UA/Alb, uric acid-to-albumin ratio.

All clinical severity scores were markedly elevated in the non-survivor group. Median qSOFA score was 3.0 (3.0–3.0) in non-survivors compared to 1.0 (0.0–2.0) in survivors (*p* < 0.001). Similarly, SOFA scores were significantly higher in non-survivors [12.0 (11.0–13.0) vs. 6.0 (4.0–8.0), *p* < 0.001]. The same pattern was observed for APACHE II [57.0 (50.0–70.0) vs. 18.0 (14.0–22.0), *p* < 0.001] and SAPS II scores [65.0 (52.0–78.0) vs. 35.0 (29.0–41.0), *p* < 0.001], indicating a substantially higher disease burden in non-survivors.

Laboratory findings demonstrated a consistent pattern reflecting increased inflammatory burden, metabolic dysregulation, and impaired nutritional status in non-survivors. Hemoglobin levels were significantly lower in non-survivors [9.9 (8.9–11.9) g/dL] compared to survivors [12.2 (10.8–13.7) g/dL, *p* < 0.001], while RDW values were significantly higher [17.0 (15.3–19.0)% vs. 14.5 (13.5–15.6)%, *p* < 0.001]. Albumin levels were markedly reduced in non-survivors [25.5 (23.0–28.7) g/L] compared to survivors [36.0 (32.0–40.0) g/L, *p* < 0.001]. In contrast, uric acid, triglyceride, and glucose levels were all significantly elevated in non-survivors (*p* < 0.001 for all comparisons), indicating increased oxidative stress and metabolic imbalance.

Derived hematological and metabolic indices further emphasized these differences. The hemoglobin-to-RDW ratio (HRR) was significantly lower in non-survivors [0.58 (0.48–0.75)] compared to survivors [0.83 (0.72–0.97), *p* < 0.001], reflecting impaired hematological homeostasis. Conversely, the RDW-to-albumin ratio (RAR) was significantly higher in non-survivors [0.66 (0.56–0.78) vs. 0.40 (0.35–0.48), *p* < 0.001]. Similarly, the TyG index and UA/Alb ratio were markedly elevated in non-survivors [9.32 (9.03–9.74) vs. 8.48 (8.23–8.73) and 0.29 (0.26–0.35) vs. 0.18 (0.15–0.21), respectively; *p* < 0.001 for both].

**Figure 1:**
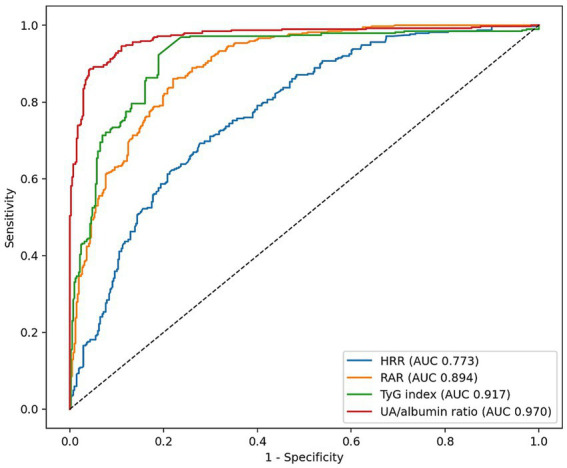
ROC curves for 28-day mortality biomarkers.

Receiver operating characteristic (ROC) curve analysis demonstrated strong discriminative performance for all evaluated biomarkers. The UA/Alb ratio showed the highest predictive accuracy with an area under the curve (AUC) of 0.968, followed by the TyG index (AUC: 0.916) and RAR (AUC: 0.900), all indicating excellent discrimination. The HRR demonstrated moderate predictive performance (AUC: 0.771). The optimal cut-off values for UA/Alb ratio, TyG index, RAR, and HRR were 0.212, 8.85, 0.495, and 0.701, respectively, with corresponding high sensitivity and specificity values (Table 2; Figure 1).

**Table 2:** Receiver operating characteristic (ROC) analysis for predicting 28-day mortality.

Variable	AUC (95% CI)	Cut-off	Sensitivity (%)	Specificity (%)	*p*-value
UA/Alb ratio	0.970 (0.957–0.979)	0.212	92.4	90.8	<0.001
TyG index	0.916 (0.898–0.934)	8.85	85.7	82.1	<0.001
RAR	0.894 (0.879–0.921)	0.495	83.6	80.5	<0.001
HRR	0.773 (0.742–0.800)	0.701	70.3	68.9	<0.001

Receiver operating characteristic (ROC) curve analysis was performed to evaluate the discriminative ability of each biomarker for predicting 28-day mortality. Optimal cut-off values were determined using the Youden index. AUC: area under the curve; CI: confidence interval.

In univariate logistic regression analysis, age, all clinical severity scores (SOFA, qSOFA, APACHE II, SAPS II), and all evaluated biomarkers (HRR, RAR, TyG index, and UA/Alb ratio) were significantly associated with 28-day mortality (all *p* < 0.001). Higher values of RAR, TyG index, and UA/Alb ratio were associated with increased mortality risk, whereas higher HRR values were associated with reduced mortality risk (Table 3A).

**Table 3:** Logistic regression analysis for 28-day mortality.

(A) Univariate analysis		
Variable	OR (95% CI)	*p*-value
Age	1.048 (1.034–1.062)	<0.001
SOFA score	1.452 (1.382–1.525)	<0.001
qSOFA score	3.215 (2.711–3.814)	<0.001
APACHE II score	1.321 (1.287–1.356)	<0.001
SAPS II score	1.118 (1.099–1.137)	<0.001
HRR	0.021 (0.010–0.045)	<0.001
RAR	18.72 (12.41–28.24)	<0.001
TyG index	3.98 (3.21–4.94)	<0.001
UA/Alb ratio	145.3 (82.6–255.4)	<0.001

(B) Multivariable analysis (final model)		
Variable	Adjusted OR (95% CI)	*p*-value
Age	1.006 (0.994–1.019)	0.334
SAPS II score	1.091 (1.070–1.113)	<0.001
HRR	0.742 (0.435–1.266)	0.275
RAR	1.842 (0.864–3.927)	0.117
TyG index	1.214 (0.691–2.134)	0.502
UA/Alb ratio	28.47 (12.96–62.51)	<0.001

Univariate and multivariable logistic regression analyses were performed to identify predictors of 28-day mortality. Variables with clinical relevance and statistical significance in univariate analysis were included in the multivariable model. To avoid multicollinearity, only SAPS II score was included among severity scores. Multivariable analysis was conducted using complete-case data (*n* = 805). OR, Odds ratio; CI, Confidence interval.

Multivariable logistic regression analysis was performed including all 805 patients, as no missing data were present in the variables included in the final model. In this model, SAPS II score and UA/Alb ratio remained statistically significant independent predictors of 28-day mortality (*p* < 0.001 for both). In contrast, age (*p* = 0.334), HRR (*p* = 0.275), RAR (*p* = 0.117), and TyG index (*p* = 0.502) were not independently associated with mortality after adjustment (Table 3B).

**Figure 2:**
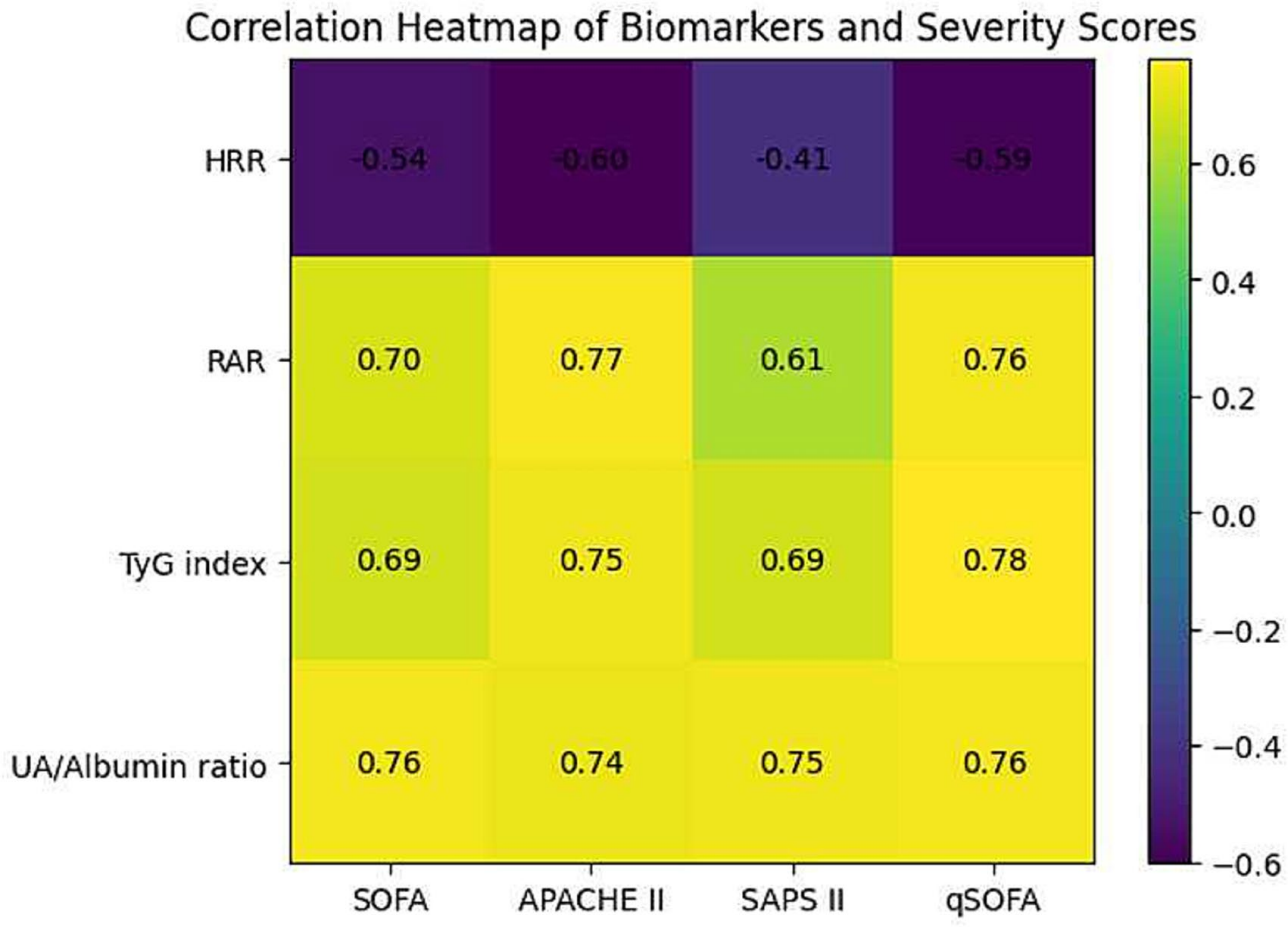
Heatmap showing correlations between hematological/metabolic indices and clinical severity scores. Spearman correlation coefficients (*ρ*) are presented. HRR demonstrated moderate negative correlations with severity scores, whereas RAR, TyG index, and UA/Alb ratio showed strong positive correlations with all severity indices (*p* < 0.001 for all).

Correlation analysis revealed that RAR, TyG index, and UA/Alb ratio were positively correlated with disease severity scores, including SOFA and SAPS II, whereas HRR demonstrated an inverse correlation with disease severity parameters, supporting the notion that these indices largely reflect the overall burden of critical illness (Table 4; Figure 2).

**Table 4:** Correlation of biomarkers with severity scores.

Biomarker	Severity score	Spearman’s ρ	*p*-value
HRR	SOFA	−0.536	<0.001
HRR	APACHE II	−0.602	<0.001
HRR	SAPS II	−0.411	<0.001
HRR	qSOFA	−0.589	<0.001
RAR	SOFA	0.698	<0.001
RAR	APACHE II	0.765	<0.001
RAR	SAPS II	0.611	<0.001
RAR	qSOFA	0.756	<0.001
TyG index	SOFA	0.685	<0.001
TyG index	APACHE II	0.752	<0.001
TyG index	SAPS II	0.688	<0.001
TyG index	qSOFA	0.781	<0.001
UA/Alb ratio	SOFA	0.755	<0.001
UA/Alb ratio	APACHE II	0.741	<0.001
UA/Alb ratio	SAPS II	0.749	<0.001
UA/Alb ratio	qSOFA	0.759	<0.001

Spearman correlation analysis was performed to evaluate the relationship between hematological/metabolic indices and disease severity scores. All correlations were statistically significant (*p* < 0.001).

Overall, non-survivors exhibited a distinct clinical and biochemical profile characterized by advanced age, higher disease severity scores, increased inflammatory and metabolic burden, and impaired nutritional and hematological status. Among all evaluated biomarkers, the UA/Alb ratio demonstrated the highest discriminative ability and independent prognostic value, highlighting its potential as a practical and powerful biomarker for early risk stratification in critically ill patients with sepsis.

## 4 Discussion

In this retrospective cohort study including 805 critically ill patients with sepsis, we demonstrated that several hematological and metabolic indices derived from routine laboratory parameters are significantly associated with 28-day mortality. Among these, the uric acid-to-albumin ratio (UA/Alb) exhibited the highest discriminative performance and remained an independent predictor of mortality after adjustment for disease severity. In contrast, HRR, RAR, and TyG index, although strongly associated with mortality in univariate analyses, did not retain independent significance in the multivariable model. UA/Alb appears to capture combined oxidative stress and nutritional impairment; however, given the substantial construct overlap with established severity scores, its incremental prognostic value remains uncertain (1–5, 19–21).

The baseline characteristics of our cohort clearly demonstrated that non-survivors had significantly higher disease severity scores, including SOFA, qSOFA, APACHE II, and SAPS II, confirming that organ dysfunction burden remains the principal determinant of mortality in sepsis (1–4). However, beyond these established clinical parameters, non-survivors also exhibited a distinct biochemical profile characterized by lower hemoglobin and albumin levels, and higher RDW, uric acid, triglyceride, and glucose levels. This constellation reflects the combined effects of systemic inflammation, oxidative stress, and metabolic dysregulation, which are central components of sepsis pathophysiology (1, 5, 13–21).

The HRR, which integrates hemoglobin and RDW, was significantly lower in non-survivors, indicating impaired oxygen delivery capacity in the setting of increased inflammatory burden. This is consistent with previous studies demonstrating that anemia of inflammation and elevated RDW are associated with adverse outcomes in critically ill patients (6–8). However, in the present study, HRR lost its independent predictive value after adjustment for SAPS II, suggesting that it primarily reflects overall disease severity rather than functioning as an independent prognostic determinant (6–8, 22, 23).

Similarly, the RAR was significantly elevated in non-survivors and demonstrated excellent discriminative performance in ROC analysis. The biological rationale of RAR lies in the combination of RDW, an indicator of erythrocyte dysfunction and inflammation, and albumin, a negative acute-phase reactant reflecting nutritional status and systemic inflammation. Although RAR showed strong associations with mortality, its lack of independence in multivariable analysis suggests that its prognostic value is largely mediated through its correlation with established severity indices (9–12, 22, 23).

The TyG index, a surrogate marker of insulin resistance and metabolic stress, was also significantly higher in non-survivors. Hyperglycemia and dyslipidemia are well-recognized features of the stress response in sepsis and have been associated with increased mortality in previous studies (13–18). However, similar to HRR and RAR, the TyG index did not remain independently associated with mortality after adjustment, indicating that it reflects metabolic consequences of severe illness rather than an independent pathogenic pathway (13–18, 24). This finding underscores that not all statistically significant biomarkers retain independent prognostic value after adjustment, emphasizing the importance of multivariable evaluation.

It is important to emphasize that the evaluated indices should be interpreted as markers of disease severity rather than causal factors contributing to mortality. These biomarkers reflect underlying pathophysiological processes, including inflammation, oxidative stress, and metabolic dysregulation, but do not represent direct therapeutic targets.

Among all evaluated biomarkers, the UA/Alb ratio demonstrated the strongest and most consistent prognostic performance. It showed high discriminative performance in ROC analysis and remained an independent predictor of 28-day mortality even after adjustment for SAPS II. This finding is biologically plausible. Uric acid is a marker of oxidative stress, tissue hypoxia, and cellular turnover, all of which are prominent in severe sepsis. In contrast, albumin has well-established antioxidant, anti-inflammatory, and endothelial stabilizing properties. Therefore, the UA/Alb ratio simultaneously captures both increased oxidative stress and reduced physiological reserve. This dual-pathway representation may explain its superior performance compared to other indices that reflect only a single aspect of disease biology (19–21, 25, 26). The very high discriminative performance of the UA/Alb ratio should be interpreted with caution given the retrospective design and potential for overfitting, as it may reflect potential overfitting or dataset-specific effects inherent to retrospective analyses.

The very high AUC observed for the UA/Alb ratio should be interpreted with caution. Internal validation using bootstrap resampling and cross-validation suggested a degree of optimism in the initial estimates, indicating potential overfitting inherent to retrospective analyses.

The correlation analysis further supports this interpretation. While RAR, TyG, and UA/Alb were positively correlated with severity scores, HRR showed an inverse relationship, indicating that these indices are closely linked to the underlying disease burden (6–21). However, the persistence of UA/Alb as an independent predictor suggests that it captures additional pathophysiological information not fully represented by conventional severity scores (19–21). The extreme odds ratio observed for HRR in univariate analysis may be influenced by scaling effects and should be interpreted cautiously.

From a clinical standpoint, the integration of simple and reproducible biomarkers into routine practice may significantly enhance early risk stratification in sepsis. The UA/Alb ratio, given its strong predictive performance and independence from conventional severity scores, appears to be a promising and clinically applicable candidate. Its ease of calculation, wide availability, and low cost make it particularly attractive for real-world clinical use, including in resource-constrained environments. Importantly, the ability to identify high-risk patients early in the disease course may facilitate timely escalation of care, optimization of hemodynamic management, and closer monitoring, which are critical determinants of outcome in sepsis. Future prospective and multicenter studies should aim to validate these findings and explore whether incorporating UA/Alb into existing prognostic models can improve clinical decision-making and patient survival (1–5, 19–21).

Taken together, our findings highlight an important conceptual distinction. Many laboratory-derived indices are significantly associated with mortality because they reflect the severity of illness; however, only a subset provide independent prognostic information beyond established clinical models. In our study, UA/Alb ratio fulfilled this criterion, whereas HRR, RAR, and TyG index did not (6–21).

## 5 Limitations

This study has several limitations. First, its retrospective design limits causal inference and is subject to potential selection bias. Second, although data cleaning and recalculation of derived indices were performed before the final analyses, the retrospective design may still have introduced information bias. Third, laboratory parameters were assessed only at ICU admission, and dynamic changes over time were not evaluated. Fourth, potential multicollinearity among severity scores was addressed by including only SAPS II in the final model; however, residual confounding cannot be entirely excluded.

Given that several components of the evaluated indices (e.g., albumin and glucose) are also incorporated into established severity scores, the observed associations may partially reflect construct overlap rather than independent biological signal. Therefore, the apparent superiority of UA/Alb should be interpreted cautiously, and no formal incremental predictive value (e.g., NRI or IDI) was demonstrated in this study.

The observed mortality rate of 71.7% is higher than typically reported in general ICU sepsis cohorts. This likely reflects the advanced age and markedly elevated disease severity scores in our population (median SOFA: 12, APACHE II: 57), indicating that our cohort represents a high-risk subgroup rather than a generalizable sepsis population.

The very high discriminative performance observed for the UA/Alb ratio should be interpreted with caution. Given the retrospective design and the absence of external validation, these findings may be partially cohort-specific. Therefore, validation in independent, prospective, and multicenter cohorts is essential before clinical implementation.

No external validation cohort was available. Therefore, the findings should be considered hypothesis-generating and require confirmation in independent populations.

Finally, the study was conducted in a limited number of centers, which may affect the generalizability of the findings.

## 6 Conclusion

In conclusion, hematological and metabolic indices derived from routine laboratory parameters are strongly associated with 28-day mortality in patients with sepsis. Among these, the UA/Alb ratio demonstrated the highest discriminative ability and remained an independent predictor of mortality, suggesting that it may serve as a simple, accessible, and clinically valuable biomarker for early risk stratification in critically ill septic patients.

## Data Availability

The raw data supporting the conclusions of this article will be made available by the authors, without undue reservation.
